# Facile Synthesis of “Boron-Doped” Carbon Dots and Their Application in Visible-Light-Driven Photocatalytic Degradation of Organic Dyes

**DOI:** 10.3390/nano10081560

**Published:** 2020-08-08

**Authors:** Zhili Peng, Yiqun Zhou, Chunyu Ji, Joel Pardo, Keenan J. Mintz, Raja R. Pandey, Charles C. Chusuei, Regina M. Graham, Guiyang Yan, Roger M. Leblanc

**Affiliations:** 1 National Center for International Research on Photoelectric and Energy Materials, School of Materials and Energy, Yunnan University, Kunming 650091, China; zhilip@ynu.edu.cn (Z.P.); jichunyu@mail.ynu.edu.cn (C.J.); 2 Department of Chemistry, University of Miami, 1301 Memorial Drive, Coral Gables, FL 33146, USA; yxz431@miami.edu (Y.Z.); joelpardo24@gmail.com (J.P.); kjm154@miami.edu (K.J.M.); 3 Department of Chemistry, Middle Tennessee State University, Murfreesboro, TN 37132, USA; RajaRam.Pandey@mtsu.edu (R.R.P.); Charles.Chusuei@mtsu.edu (C.C.C.); 4 Department of Neurological Surgery, Miller School of Medicine, University of Miami, Miami, FL 33136, USA; rgraham@med.miami.edu; 5 Key Laboratory of Green Energy and Environment Catalysis (Ningde Normal University), Fujian Province University, Ningde 352100, China; 6 Fujian Provincial Key Laboratory of Featured Biochemical and Chemical Materials, Ningde Normal University, Ningde 352100, China

**Keywords:** carbon dots, photo catalysis, dye degradation, environment clean, rhodamine B, methylene blue

## Abstract

Carbon dots (C-dots) were facilely fabricated via a hydrothermal method and fully characterized. Our study shows that the as-synthesized C-dots are nontoxic, negatively charged spherical particles (average diameter 4.7 nm) with excellent water dispersion ability. Furthermore, the C-dots have a rich presence of surface functionalities such as hydroxyls and carboxyls as well as amines. The significance of the C-dots as highly efficient photocatalysts for rhodamine B (RhB) and methylene blue (MB) degradation was explored. The C-dots demonstrate excellent photocatalytic activity, achieving 100% of RhB and MB degradation within 170 min. The degradation rate constants for RhB and MB were 1.8 × 10^−2^ and 2.4 × 10^−2^ min^−1^, respectively. The photocatalytic degradation performances of the C-dots are comparable to those metal-based photocatalysts and generally better than previously reported C-dots photocatalysts. Collectively considering the excellent photocatalytic activity toward organic dye degradation, as well as the fact that they are facilely synthesized with no need of further doping, compositing, and tedious purification and separation, the C-dots fabricated in this work are demonstrated to be a promising alternative for pollutant degradation and environment protection.

## 1. Introduction

The contamination of water bodies by organic dyes has quickly become a worldwide environmental concern [1]. Organic dyes are by-products of textile, printing, and plastic industries, which could damage aquatic ecosystems by suppressing light penetration and affecting photosynthesis. Furthermore, they can also lead to serious health issues such as cancer, birth defects, as well as hormone imbalances if ingested [2,3]. Unfortunately, the effective degradation of these dyes is rather difficult with traditional techniques due to their wide spreading and low concentration, as well as stable chemical nature. As a result, there is a key interest and necessity to seek innovative and viable approaches that could degrade organic dyes with high efficiency and practicality. Among the various approaches, photocatalytic dye degradation has emerged as one of the most efficient, practical, clean, and cost-effective methods, which only requires photocatalysts and light irradiation [4].

Nanomaterials derived from TiO_2_ have been regarded as promising photocatalysts for hydrogen generation via water splitting and environmental purification since the ground-breaking report by Honda and Fujishima in 1972 [5]. Especially after Carey et al. reported the first use of nano-TiO_2_ to degrade polychlorobiphenyls under ultraviolet (UV) irradiation in 1976 [6], various metal oxide-based photocatalysts, including TiO_2_ [7,8,9,10,11,12], ZnO [13,14,15,16], Bi_2_O_3_ [17,18,19], Cu_2_O [20,21], Fe_2_O_3_ [22,23], and NiO/Ni(OH)_2_ [24,25] have been developed for the photocatalytic degradation of organic dyes and pollutants. However, most of the catalysts were metal-based, which pose a potential risk of secondary pollution to water bodies [26]. In addition, these catalysts often require multi-step synthesis, which is time-consuming and cost demanding [27,28,29,30,31]. Most importantly, most of the metal-based catalysts, due to their relatively large band gaps, can only utilize UV light that only accounts for a tiny fraction of the sunlight irradiation [32]. As such, these shortcomings have significantly limited the practical applications of metal-based photocatalysts. Therefore, much effort has been devoted for developing novel photocatalysts that are nontoxic, stable, able to utilize visible light, and have facile synthesis.

Carbon dots (C-dots) are the newest member of the renowned carbon-based nanomaterials family. Since their discovery in the early 2000s, they have been regarded as the green alternatives of the traditional, semiconductor-based quantum dots (QDs) with the most potential [33,34,35,36]. In the past decades, C-dots have been widely applied in various fields including chemistry, physics, engineering and biomedicine due to their superior properties such as high photoluminescence (PL) and stability, low toxicity, high biocompatibility, excellent water dispersion ability, as well as rich and tunable surface functionalities [37,38,39,40,41,42]. Even though the exact PL mechanism of C-dots is still under debate [43,44], researchers have drawn the general conclusion that C-dots could be regarded as semiconductors that could generate electrons and holes under light irradiation. Due to the wide presence of tunable surface functionalities (i.e., surface states), the band gaps of C-dots are not locked strictly based on the energy difference of valence and conduction bands; instead, the band gaps could be tuned by the energy differences between the surface states and the valence band [45]. As a result, the potential applications of C-dots in driving photocatalytic redox reactions have been heavily investigated [46]. Unfortunately, C-dos alone have shown little activity toward the photocatalytic degradation of organic dyes [47,48,49,50,51,52]. To realize the efficient degradation of organic dyes, C-dots have to be integrated in metal-based heterostructures [50,51,52,53,54,55,56,57]. Recently, some encouraging reports demonstrated the efficient photocatalytic degradation of organic dyes by C-dots alone [58,59,60,61,62,63,64]; however, various factors including limited light absorption, extra surface doping, and tedious separation significantly limited their practical applications. 

Herein, in this paper, we report a simple hydrothermal method for the fabrication of C-dots from citric acid (CA) and 1,2-diboranyethane (DBE). The C-dots are facilely synthesized in one-step, nontoxic, and highly photostable. Most importantly, they demonstrate excellent photocatalytic activity toward the degradation of methylene blue (MB) and rhodamine B (RhB) under visible light irradiation. The determined dye degradation efficiency and rate constants catalyzed by this C-dots alone are comparable to those metal-based heterostructures and generally better than other C-dots photocatalysts reported in the literature. The C-dots prepared in this work are shown to be a very promising alternative for dye degradation and environment cleanliness.

## 2. Materials and Methods 

### 2.1. Chemicals and Materials 

Citric acid, 1, 2-diboranyethane, rhodamine B, methylene blue, and N, N-dimethylformamide (DMF, anhydrous, 99.8%) were purchased from Sigma-Aldrich (St. Louis, TX, USA). Argon gas was bought from Airgas (Miami, FL, USA). All chemicals were used as received without further purification unless otherwise noted. Purified water, with a surface tension of 72.6 mN·m^−1^ and a resistivity of 18 MΩ·cm at 20.0 ± 0.5 °C, was obtained from a Modulab 2020 water purification system (San Antonio, TX, USA). Dialysis bags with a molecular weight cutoff (MWCO) of 1000 were purchased from Thermo Scientific (Rockford, TX, USA). CellTiter 96 Aqueous One Solution Cell Proliferation Assay (MTS) was obtained from Promega (Madison, WI, USA). 

### 2.2. Synthesis of C-Dots 

The C-dots used in this study were prepared via a hydrothermal method. Briefly, 1 g of citric acid was quickly transferred to a Teflon-lined autoclave (50 mL); then 1, 2-diboranyethane (0.3 g) was also quickly added, and after that, 20 mL of anhydrous DMF were introduced into the mixture. All of the above steps were carried out under argon gas protection. The autoclave was purged with argon gas for 5 min, and then it was sealed and heated at 160 °C for 6 h. After that, it was allowed to cool down naturally. The resulted reaction mixture was centrifuged for 15 min at 3000 rpm to remove any large particles; then, the supernatant was subjected to dialysis with a semi membrane dialysis bag (MWCO 1000) against pure water for 3 days, with the water changed every 4 h during the day. C-dots in brownish powder form were obtained after the removal of water.

### 2.3. Characterizations of C-Dots

UV-Vis absorption spectra were obtained using a Cary 100 UV-Vis spectrophotometer (Agilent Technologies, Santa Clara, CA, USA) with a 1 cm optical path length cell; the absorption of C-dots was measured using an aqueous (deionized water) solution of C-dots at 1.25 mg/mL. Fourier-transform infrared (FTIR) spectra were taken with a PerkinElmer FTIR (Frontier, Waltham, MA, USA) spectrometer using the attenuated total reflection (ATR) technique with air as background, and about 5 mg of powder C-dots were used, which were recovered later on. Fluorescence spectra were measured by a fluorescence spectrophotometer (Horiba Jobin Yvon Fluorolog–3, Kyoto, Japan) at 25 °C with a slit width of 5 nm for both excitation and emission, an aqueous (deionized water) solution of C-dots at 1.25 mg/mL was used for the measurements. To evaluate the photostability of our C-dots under ambient light, C-dots solutions (1.25 mg/mL) was placed on a bench top exposed to room light. Their fluorescence emissions were tested at different intervals (1, 3, 7, 14, 21, 30, 60, and 90 days). X-ray photoelectron spectra (XPS) were acquired using a Perkin-Elmer PHI (Waltham, MA, USA) 560 system with a double-pass cylindrical mirror analyzer operated using a Mg Kα anode with an h*ν* = 1253.6 eV photon energy operated at 250 Watts and 13 kV. Roughly 8 mg of “B-doped” C-dots samples were mounted as a paste onto a custom Ta foil sample holder and inserted into the XPS system via a turbopumped antechamber. Transmission electron microscopy (TEM) was performed on a JEOL 1200X TEM (Tokyo, Japan). Samples for TEM measurements were prepared as follows: C-dots were dissolved in deionized water to make a 0.1 mg/mL solution, and then the solution was sonicated for 30 min before a small drop of the solution was put onto a carbon-coated 200 mesh copper grid (EM Sciences Catalog #CF200-CU). Then, the grid containing the drop of sample was put in a small Petri dish on a small piece of parafilm and allowed to dry overnight before it was used for measurement. Atomic force microscopy (AFM) was obtained on an Agilent 5420 atomic force microscope (Santa Clara, CA, USA) with the tapping mode. The samples were prepared as follows: C-dots were dissolved in deionized water to make a 0.1 mg/mL solution; then, the solution was sonicated for 30 min before a small drop of the solution was put onto a sample slide. Then, 3–5 drops of water were carefully dropped to the silica mica slide to dilute the sample further. Then, the slide containing the diluted sample was put in a small Petri dish on a small piece of parafilm and allowed to dry overnight before it was used for measurement. The zeta potential of the aqueous dispersion of the samples (1.25 mg/mL) were analyzed using a Zetasizer Nano ZS System (Malvern, Inc., Malvern, UK) with irradiation from a standard 633 nm laser at 25 °C. 

### 2.4. C-Dots Quantum Yield Determination

The quantum yield was calculated based on the following equation (the relative method):

(1) $$\Phi =\Phi_{S}\;\times \;\frac{I}{I_{S}}\;\times \;\frac{A_{S}}{A}\;\times \;\frac{n^{2}}{n_{S}^{2}}\;\times \;\frac{D}{D_{S}}$$

where *Φ_s_* is the quantum yield of the standard sample; *I_S_* and *I* are the integrated area under the emission peaks of the standard and unknown samples, respectively; *A_S_* and *A* are the absorbance of the excitation wavelengths of the standard and unknown samples, respectively; n_S_ and n are the refractive indices of the standard and unknown samples, respectively; and *D/D_S_* is the dilution ratio if the samples are diluted during the measurement of fluorescence emission. In this experiment, UV-Vis absorption and the fluorescence emission spectra of C-dots were recorded using deionized water as the solvent. Quinine sulfate in 0.1 M H_2_SO_4_ (quantum yield: 0.54) was used as the standard for quantum yield calculation. The refractive index of water is 1.333 and for 0.1 M H_2_SO_4_, it is 1.346.

### 2.5. Cell Viability/Proliferation Assay 

The viability of mesenchymal stem cells (non-cancer cells) was determined based on the MTS assay as previously described [65,66]. Briefly, 24 h prior to C-dots treatment, 5000–10,000 cells per well were plated into 96-well plates. Subsequently, the cells were treated with different concentrations (0.0, 0.1, 1, 10, and 50 µg/mL) of C-dots. The cell viability was analyzed after 72 h exposure to the C-dots using the CellTiter 96^®^ Aqueous One Solution Cell Proliferation Assay according to the manufacturer’s instruction. Experiments were repeated at least three times, and data are presented as the average of the separate experiments. Viability was calculated as a percentage of the non-treated cells (i.e., concentration of C-dots is 0 µg/mL).

### 2.6. Photocatalytic Degradation of Organic Dyes

RhB and MB are selected as model dyes to evaluate the photocatalytic ability of our C-dots in this study. Briefly, 3 mg of C-dots and 0.2 mL of aqueous RhB (10 mg/L) solution were mixed, and then 3.8 mL of deionized water were added. The resulted mixture (final concentration of RhB, 0.5 mg/L) was transferred to an UV-Vis cuvette and ultrasonicated for 2 min to disperse the C-dots under dark. Then, the cuvette, vigorously stirred at 200 rpm, was placed in front of the solar simulator (Oriel Instruments, Newport Corporation) with high power mercury-xenon light source set at 310 W (see Supporting Information, Figure S1 for the spectrum of the lamps used). The UV-Vis absorption spectra of RhB were checked every 10 min under irradiation to monitor the degradation of RhB. Similar procedures were carried out for MB except that the amount of MB used was different. Instead of 0.2 mL, 1.0 mL of aqueous MB (10 mg/L) solution was mixed with 3 mg of C-dots. As a result, only 3 mL of deionized water was used later to make the desired mixture solution for test (final concentration of MB, 2.5 mg/L). Same procedures as for RhB were carried out to study and monitor the photocatalyzed degradation of MB by C-dots under light irradiation.

## 3. Results

The fabrication of C-dots from CA and ethylenediamine (EDA) is a very classical, facile bottom–up approach to prepare C-dots, in which CA is considered as the main carbon precursor and EDA is the nitrogen-doping agent [67,68]. To explore the potential effects of boron doping on the properties of this type of C-dots [69,70], we replaced EDA with DBE in the synthesis, in which the nitrogen atoms were replaced with boron atoms (Supporting Information, Figure S2). Excitingly, hydrothermal treatment of CA and DBE at 160 °C for 6 h produced the desired C-dots (herein named as “B-doped” C-dots). The reaction was carried out with anhydrous DMF as solvent in an argon atmosphere in order to minimize the hydrolysis of DBE, which is prone to hydrolysis due to the presence of borane functions. Interestingly, N-doped C-dots prepared from CA and EDA are a colorless gel-like form [67], while the “B-doped” C-dots in this study are brownish powder. To further analyze the morphological, physical, and chemical properties, characterizations of the “B-doped” C-dots by TEM and AFM microscopy, UV-Vis, FTIR, fluorescence, and XPS spectroscopy, as well as zeta potential were performed.

**Figure 1:**
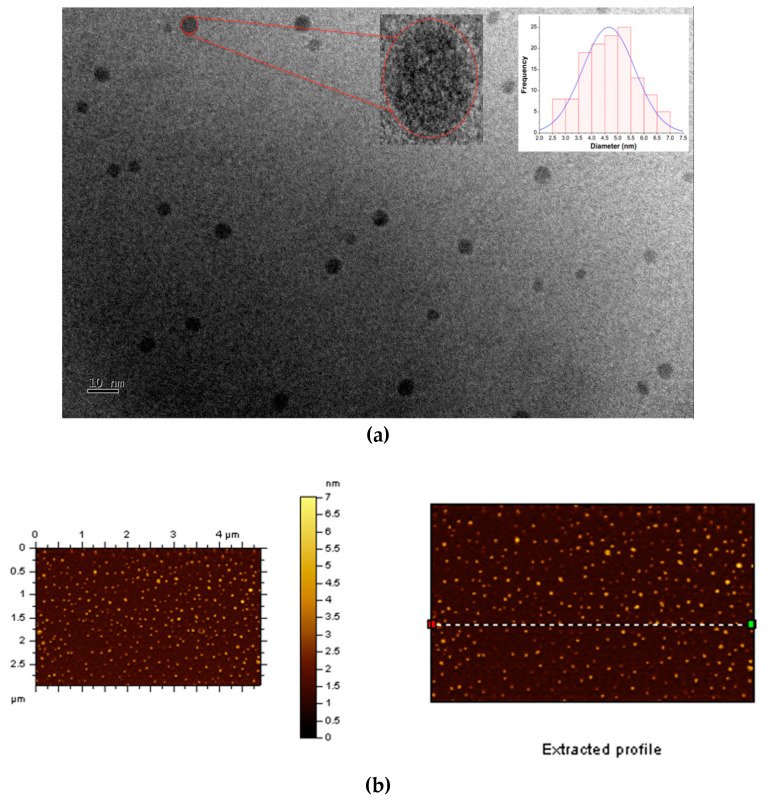

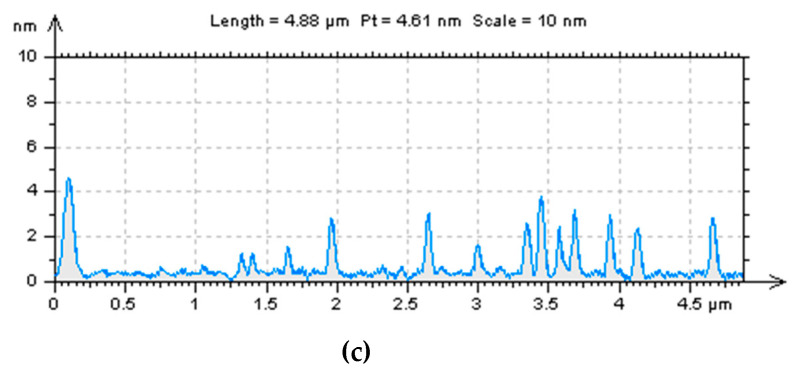
Morphological characterizations of “B-doped” C-dots: (**a**) TEM image of C-dots, the left inset is the enlarged image of one particle and the right inset is the size distribution histogram of C-dots; (**b**) atomic force microscopy (AFM) image of C-dots; (**c**) height profile of C-dots corresponding the profile line in Figure 1b.

TEM and AFM microscopy were first applied to explore the morphologies and structural characteristics of the “B-doped” C-dots. The TEM image of C-dots (Figure 1A) shows that they are well-dispersed spherical particles with no obvious agglomerates. There are no noticeable lattices in these C-dots (Figure 1a, left inset), indicating that they have amorphous cores, instead of graphitic ones. These particles range from 2.5 to 7.0 nm in diameters with an average of 4.7 nm (Figure 1a, right inset). As shown in AFM images (Figure 1b), these particles are also evenly distributed with no noticeable agglomerates; the spherical particles are about 2.5–5.0 nm in height (Figure 1c), which is consistent with the diameter distributions obtained from TEM images. In summary, microscopic studies show that the “B-doped” C-dots (average diameter 4.7 nm) are slightly larger in size than their counterparts synthesized from CA and EDA (average diameter 3.0 nm) [71].

**Figure 2:**
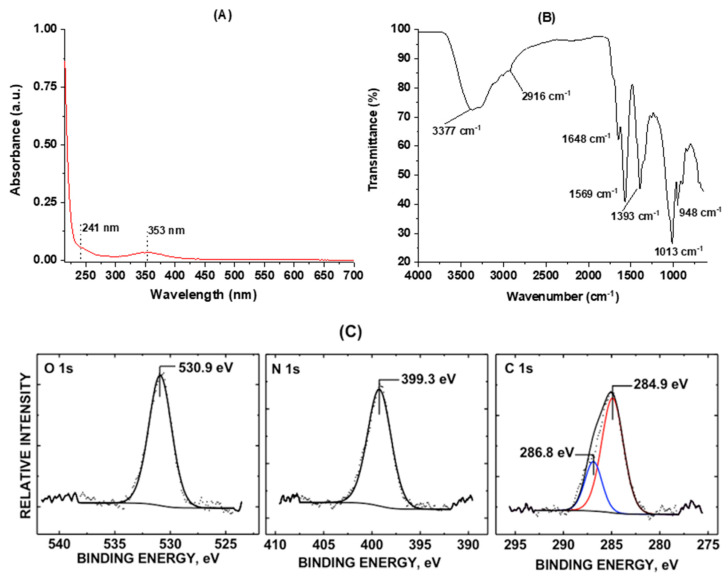
Spectroscopic characterization of the “B-doped” C-dots: (**A**) UV-Vis absorption spectrum of C-dots; (**B**) Fourier-transform infrared (FTIR) spectrum of C-dots with air as background; (**C**) XPS core levels of O 1s, N 1s, and C 1s orbitals of C-dots.

Spectroscopic characterization techniques such as UV-Vis, FTIR, fluorescence, and XPS were then applied to explore the functionalities, chemical compositions, and PL behaviors of the “B-doped” C-dots. The UV-Vis absorption spectrum (Figure 2a) displays two typical peaks at 241 and 353 nm, which corresponds to π–π* and n–π* transitions, indicating the presence of C=C and C=O bonds in the conjugate structures, respectively [67,72]. To further explore the functionalities, FTIR spectroscopy equipped with ATR accessories was performed. In the FTIR spectroscopy (Figure 2B), various functional groups such as OH and NH (3377–3250 cm^−1^), CH (2916 cm^−1^), C=O (1648 cm^−1^), C=C (1569 cm^−1^), C–H (1393 cm^−1^), C–O (1013 cm^−1^), and O–H (948 cm^−1^) are observed, indicating the rich presence of functionalities such as hydroxyls, amines, and carbonyls as well as carboxyls [72,73]. These functionalities are further confirmed by XPS spectroscopy, which reveals 75.8% carbon, 19.1% oxygen and 5.1% nitrogen on the C-dots surface (Supporting Information, Table S1). 

It is worth mentioning that we did not observe any B 1s core level signal in the XPS spectroscopy after several attempts. Interestingly, similar observations have been reported, in which some doping elements were not found in the “doped” C-dots. For instance, in Nabid and co-workers’ report [74], boron was hardly found in C-dots prepared from hydrothermal treatment at 160 °C using citric acid monohydrate as the carbon source, dicyandiamide and boric acid as nitrogen and boron-doping agents, respectively. In a different study, Wu and co-workers reported that the addition of varied metal ions during the hydrothermal treatment of *p*-phenylenediamine led to the formation of fluorescent C-dots with emission wavelengths up to 700 nm [75]. Strikingly, although metal ions played a crucial role in the synthesis of C-dots with varied quantum yields, they were absent in the formed C-dots; that is, the obtained C-dots were metal-free, and the metal ions played a role similar to a “catalyst” during the C-dots formation. In light of these reports, it might be possible that a similar process has happened in our synthesis; that is, the doping element “boron” played a similar role as a “catalyst” during the formation of C-dots. As a result, we barely observed any boron in the C-dots formed, although boron was meant to be the doping element. Furthermore, we attribute the nitrogen (5.1%) observed in our XPS characterization to the contribution of the solvent we used. Although our synthesis agents (citric acid and 1,2-diboranyethane) do not contain any nitrogen, the solvent (dimethylformamide, DMF) we used contains a fairly high percentage of nitrogen (about 19.2%), and DMF was used in an excess amount (20 mL of DMF was used with only 1 g of citric acid and 0.3 g of 1,2-diboranyethane). The phenomena in which solvents took part in the formation of C-dots have been frequently reported in the literature recently. For example, in the investigations of polyethylene glycol (PEG)-assisted synthesis of metal NPs, Feldmann and co-workers frequently observed relatively intense emissions of blue light in their products that were hard to explain [76]. The unexpected fluorescence was later on attributed to C-dots formed from the thermal decomposition of solvent PEGs during metal NPs synthesis [77].

Figure 2C shows stackplots for the O 1s, N 1s, and C 1s core levels of “B-doped” C-dots scanned. The C 1s oxidation state at 286.8 eV is consistent with a C–O–H bond within the sample surface [71]. The O 1s oxidation state at 530.9 eV is indicative of C–O and C–N bonded groups to the C-dots, which is also observed by Kumar et al. [78]. The binding energy peak center at 399.3 eV, which is observed in this sample, is indicative of the presence of amine (–NH_2_) groups [79]. Based on the relative, deconvoluted peak area ratios of the C 1s level, the percentages of C=C and C–O/C–N carbon are 74.7% and 25.3%, respectively. In summary, the spectroscopic characterizations indicate a rich presence of surface functionalities such as hydroxyl and carboxyl groups. Further, the zeta potential shows that the “B-doped” C-dots are slightly negatively charged with a value of −11.6, which corresponds well with the spectroscopic study of the surface functionalities. The negative zeta potential value also partially explains the good dispersibility of C-dots observed in the microscopic studies discussed above.

**Figure 3:**
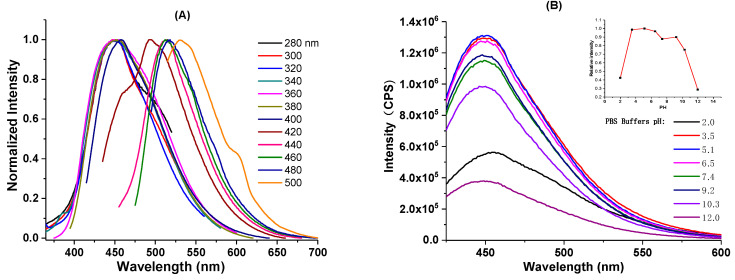
(**A**) Normalized emission spectra of C-dots; (**B**) fluorescence emission spectra of C-dots excited at 360 nm under different pH conditions (the inset shows the relative fluorescence intensity of C-dots excited at 360 nm under different pH).

Next, we investigated the PL behavior of the obtained C-dots. Excitation-wavelength-dependent PL emission is among the most notable characteristics of C-dots. As seen from the fluorescence emission spectrum (supporting information, Figure S3a), the as-prepared C-dots do demonstrate such behavior with the maximum emission at 450 nm when excited at 360 nm. The excitation-wavelength-dependent PL behavior is more obvious when the emission spectrum is normalized. When excited between 280 and 380 nm, the emissions stay around 450 nm, while the emission spectra gradually red shift as the excitation wavelengths increase. The emission peak eventually shifts to 550 nm when excited at 500 nm (Figure 3a). As can be seen, the PL behavior of the “B-doped” C-dots is similar to the typical optical properties often observed from citric acid-based C-dots prepared from the bottom–up approach [80,81]. The PL of this type of C-dots could be explained by mainly two mechanisms, namely molecular state and carbon core state [44]. The typical absorption peaks at 340–350 nm and their corresponding emissions around 450 nm often observed in those citric acid-based bottom–up C-dots are believed to be related to the fluorescent molecular species (i.e., citrazinic acid type derivatives), which are generated together with the formation of C-dots [82,83,84]. The carbon core state, on the other hand, is related to the aromatic domains formed after extended heating or reaction duration, in which the molecular fluorophores likely act as the seeds for the formation of aromatic domains [73]. Since molecular fluorophores could be trapped inside C-dots or physically absorbed on the surface of C-dots, purification techniques (i.e., dialysis or size-exclusion chromatography) that could reduce the contents of molecular fluorophores on the C-dots might help reduce the complexity of the C-dots’ optical properties [80].

Next, we investigated the effects of pH on the PL property of the C-dots. Encouragingly, our C-dots were found to be stable in both acidic and basic conditions (pH: 3.5 to 10.3), with the emission wavelengths essentially unmoved. In strong acidic (pH = 2.0) or basic (pH = 12.0) conditions, wavelengths of the emission were slightly shifted; however, intensities were significantly decreased (Figure 3b). The relatively high pH stability of our C-dots is in contrast to some C-dots that are highly pH-sensitive [85,86,87], and this property endows our C-dots with the ability to adapt to different water environments (i.e., different pH) as photocatalysts. Photostability is an important factor to consider when molecular fluorophores or nanomaterials are applied for photocatalysis, bioimaging, and optoelectronics; thus, we also studied the PL stability of the “B-doped” C-dots under ambient light. As can be seen, the PL emission intensity of C-dots stays essentially the same after 3 months (Supporting Information, Figure S3b), demonstrating the high stability of the as-synthesized C-dots. In addition, according to Equation (1) presented in Section 2.4, the fluorescence quantum yield of our C-dots, using quinine sulfate dissolved in 0.1 M H_2_SO_4_ as the standard, was determined to be 11%. Last, according to the cell viability test, the “B-doped” C-dots demonstrated no observable cytotoxicity even when used in high concentrations (Supporting Information, Figure S4). Collectively considering these excellent properties, “B-doped” C-dots could be excellent materials for environment-related (i.e., photocatalytic dye degradation) applications.

**Figure 4:**
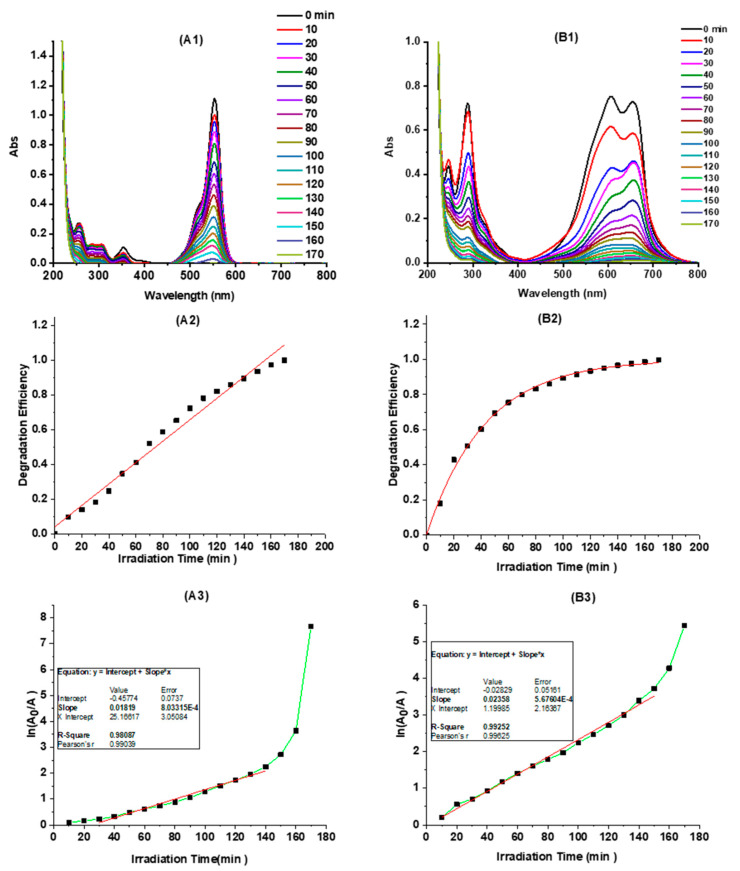
The photocatalytic degradation of rhodamine B (RhB) and methylene blue (MB) catalyzed by C-dots (0.75 mg/mL): the UV-Vis absorption spectra of RhB (**A1**) and MB (**B1**) at different irradiation times; the degradation efficiency (%) of RhB (**A2**) and MB (**B2**) by monitoring the absorption changes at 554 nm and 605 nm, respectively, at different irradiation times; irradiation-time-dependent pseudo first-order kinetics plots (ln(*A*_0_/*A*) vs. irradiation time) of RhB (**A3**) and MB (**B3**).

To evaluate the photocatalytic activity of the “B-doped” C-dots, two organic dyes, namely RhB and MB were selected. There are mainly two reasons to select these two dyes. First, the degradation of these two dyes has been widely used as model reactions to probe the photocatalytic ability of various materials including metal and oxide nanoparticles, quantum dots, and carbon-based nanomaterials [88,89,90]. Second, these two dyes are both positively charged, while our C-dots have a negative zeta potential (−11.6). As a result, the photocatalytic property of C-dots was evaluated by the degradation of RhB and MB in the presence of 0.75 mg/mL of C-dots under simulated sunlight. As can be seen, the C-dots demonstrated excellent photocatalytic ability; both RhB and MB were quickly degraded with light irradiation, as monitored by the UV-Vis absorption spectra of the two dyes (Figure 4A1,B1). The dye degradation efficiency (or degree of degradation) could be reflected by the UV-Vis absorption changes of the dyes and calculated according to the following equation:

(2) $$D=\frac{A_{0}-A}{A_{0}}\times 100\%$$

where *A*_0_ represents the absorbance of dye at time zero, and *A* is the absorbance of dye after degradation. According to Equation (2), the degradation efficiency (%) of RhB and MB in the presence of C-dots versus irradiation time was plotted (Figure 4A2,B2). As presented, C-dots exhibited substantial photocatalytic activity in the degradation of both RhB and MB: within 60 min, RhB and MB degraded (as observed by dye absorption peaks at 554 and 605 nm, respectively) 46% and 76%, respectively, while the two dyes degraded only 28% and 60% without the presence of C-dots, respectively (Supporting Information, Figure S5). Thus, the degradation efficiency of RhB and MB are increased 64% and 27%, respectively, in the presence of “B-doped” C-dots as photocatalysts. Furthermore, both RhB and MB degraded completely within 170 min. 

According to the Langmuir–Hinshelwood dynamic model [91], the degradation kinetics of organic dyes (i.e., RhB and MB) could be simplified according to the pseudo first-order kinetic equation below if the dye solution is very dilute:

(3) $$\ln\frac{A_{0}}{A}=kt$$

where *A_0_* and *A* should be the equilibrium concentration of dye adsorption and the concentration of dye after irradiation time *t*, respectively. For calculation purposes, here, *A_0_* and *A* are taken as the absorbance of the dyes at time zero and the absorbance of the dyes after degradation time *t*, respectively; k stands for the dye degradation rate constant. According to Equation (3), the pseudo first-order degradations of RhB and MB were plotted (Figure 4A3,B3). As can be seen, both the photocatalytic degradations of RhB and MB are fitted very well with the pseudo first-order kinetics. The degradation rate constants of RhB and MB are determined to be 1.8 × 10^−2^ min^−1^ and 2.4 × 10^−2^ min^−1^, respectively. It’s worth mentioning that the degradation efficiency and rate constant of MB as monitored by the absorbance at 664 nm were also calculated, which are, as expected, very similar to those determined by using the absorbance at 605 nm (Supporting Information, Figure S6).

As such, our C-dots exhibited excellent photocatalytic activity compared to most of the bare C-dots previously reported (Table 1). Most of the C-dots previously synthesized have shown little photocatalytic activity toward dye degradation (Table 1, entries 1–4) [48,49,50,51,52]; in order to catalyze the degradation of organic dyes efficiently, they have to composite with metal-based nanoparticles (Table 1, entries 2–4) [50,51,52,53,54,55,56,57], which not only significantly complicated the preparation procedures and reduced yields, but also increased cost and environmental concerns. For example, Yu et al. reported C-dots/TNS and C-dots/P25 composites with excellent photocatalytic activity for the degradation of RhB; however, there was no activity when C-dots were used alone [52]. Recently, some encouraging studies have shown that C-dots alone could possess good to excellent photocatalytic activity toward the degradation of organic dyes (Table 1, entries 5–10); however, various factors including limited light absorption, extra surface doping, as well as tedious separation have limited the practical applications of C-dots in these studies [58,59,60,61,62,63]. For instance, C-dots prepared by Srivastava and co-workers could only utilize light with a specific wavelength for the catalytic degradation of MB [59]. In the report by Kang and co-workers, nitrogen doping is required in order to improve the photocatalytic activity; bare C-dots exhibited very low efficiency, with only 30% of methylene orange degradation after 120 min irradiation [60]. In a recent study by Leblanc and co-workers, only one fraction of their as-prepared C-dots demonstrated excellent photocatalytic activity toward the degradation of RhB and MB, which had to be separated through a tedious size exclusion chromatography [63]. Considering the excellent photocatalytic efficiency toward the degradation of RhB and MB, as well as the fact that they are facilely synthesized with no need of further doping, compositing, and tedious purification and separation, C-dots prepared in this work are shown to be a very promising alternative for dye degradation and environment cleanliness (Table 1, entry 11).

**Figure 5:**
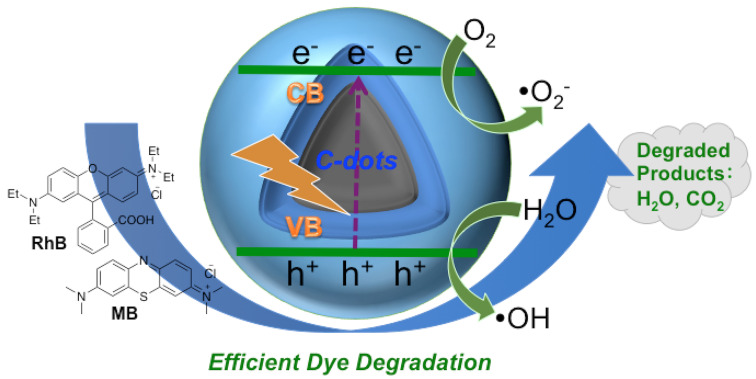
A plausible mechanism of the photocatalytic degradation of RhB and MB catalyzed by “B-doped” C-dots.

According to the results and discussions above, a plausible photocatalytic mechanism for dye degradation under the catalysis of “B-doped” C-dots is proposed (Figure 5). When photons of appropriate energy excite C-dots, electrons were excited from the ground state (valence band) to excitation state (conduction band), generating excess electrons (e^−^) and holes (h^+^). Due to the rich presence of surface defects (i.e., surface functionalities) on C-dots, some of the excited carriers are trapped, and the recombination of e^-^ and h^+^ are hindered. As a result, the organic dyes could be oxidized by the h^+^ directly to cause the degradation, which is well known [62]. In the meantime, some of the e^−^ could be captured by oxygen dissolved in solution, forming super oxide radicals (i.e., •O_2_^−^); some of the h^+^ could interact with surface-adsorbed H_2_O to form hydroxyl radicals (i.e., •OH). Reactive oxygen species (ROS) such as super oxide •O_2_^−^ [63] and hydroxyl radical •OH [62,92] are known to degrade organic dyes. 

## 4. Conclusions

The significant potential of C-dots as highly efficient photocatalysts for organic dye degradation in water remediation was explored. The visible-light-sensitive C-dots were synthesized through a facile hydrothermal method from CA and DBE, and they could be readily purified by simple dialysis. The nontoxic C-dots demonstrate typical excitation-wavelength-dependent emission with high photostability. Under visible light irradiation, the C-dots present excellent photocatalytic activity, achieving 100% of RhB and MB degradation within 170 min. The degradation of RhB and MB were well fitted with the pseudo first-order kinetics and the degradation rate constants for RhB and MB were 1.8 × 10^−2^ min^−1^ and 2.4 × 10^−2^ min^−1^, respectively. The photocatalytic degradation performances are comparable to those metal-based photocatalysts and generally better than previously reported C-dots photocatalysts. Collectively considering their excellent photocatalytic activity toward the organic dye degradation, as well as the fact that they are facilely synthesized with no need of further doping, compositing, and tedious purification and separation, C-dots fabricated in this work are shown to be a promising alternative for pollutant degradation and environment protection.

## Figures and Tables

**Table 1:** Comparison of the photo degradation performance of different C-dots-related catalysts.

Entry	Material ^c^	Dye ^d^	Degradation Efficiency	Rate Constant (min^−1^)	Reference
1	C-dots	MB	90%/1800 min;85%/120 min	2.7 × 10^−3^1.4 × 10^−2^	[48,49]
2	C-dots/ZnPor	MB	95%/60 min	-	[50]
	C-dots only		4%/60 min		
3	C-dots/TiO_2_	RhB	95%/30 min	1.1 × 10^−1^	[51]
	C-dots only		0%/30 min	5.1 × 10^−4^	
4	C-dots/TNS	RhB	95%/60 min	-	[52]
	C-dots only		0%/60 min		
5	C-dots	RhB	97%/240 min	2.5 × 10^−2^	[58]
6 ^a^	C-dots	MB	80%/60 min	2.4 × 10^−2^	[59]
7 ^b^	C-dots	MO	90%/120 min	-	[60]
8	C-dots	MB	96%/90 min	3.1 × 10^−2^	[61]
9	C-dots	MB	99.5%/130 min	3.9 × 10^−2^	[62]
10	C-dots	RhB	50%/60 min	1.3 × 10^−2^	[63]
		MB	90%/60 min	3.6 × 10^−2^	
11	C-dots	RhB	99.9%/170 min	1.8 × 10^−2^	This work
		MB	99.9%/170 min	2.4 × 10^−2^	

^a^ Single-wavelength light, instead of sunlight, was used for catalytic degradation. ^b^ Data shown for N-doped C-dots; C-dots without doping: only 31.5% dyes were degraded within 120 min. ^c^ C-dots/ZnPor: nanocomposite of zinc porphyrin functionalized graphene quantum dots; C-dots/TNS: nanocomposites of C-dots and TiO_2_ nanosheets. ^d^ MB: methylene blue; RhB: rhodamine B; MO: methylene orange.

## Supplementary Materials

The following are available online at https://www.mdpi.com/2079-4991/10/8/1560/s1, Figure S1: Spectrum of high power Mercury-Xenon Hg (Xe) lamps used in this study. Figure S2: Schematic illustration of C-dots synthesis. Figure S3: Fluorescence emission spectra of C-dots excited at different wavelengths; and relative fluorescence intensity of C-dots exposed to ambient light with different time durations. Figure S4: Cell viability test of C-dots using MTS assay. Figure S5: The photo-degradation of RhB and MB without the presence of photocatalysts. Figure S6: The photocatalytic degradation of MB catalyzed by C-dots (0.75 mg/mL) by monitoring the absorption at 664 nm. Table S1: Table showing the XPS elemental atom % and BEs of O 1s, N 1s and C 1s core levels.
