# Supplementary material for: Facile Synthesis of “Boron-Doped” Carbon Dots and Their Application in Visible-Light-Driven Photocatalytic Degradation of Organic Dyes
Source: Nanomaterials (Basel). 2020 Aug 8;10(8):1560. doi: 10.3390/nano10081560 (PMC7466398; doi:10.3390/nano10081560)
Supplement: Supplementary file 1 [file nanomaterials-10-01560-s001.pdf]

Facile Synthesis of "Boron-Doped" Carbon Dots and their Application in Visible-Light-Driven Photocatalytic Degradation of Organic Dyes

Zhili Peng [1,†], Yiqun Zhou [2,†], Chunyu Ji [1], Joel Pardo[2], Keenan J. Mintz [2], Raja R. Pandey [3], Charles C. Chusuei [3], Regina M. Graham [4], Guiyang Yan [5,6,*] and Roger M. Leblanc [2,*]

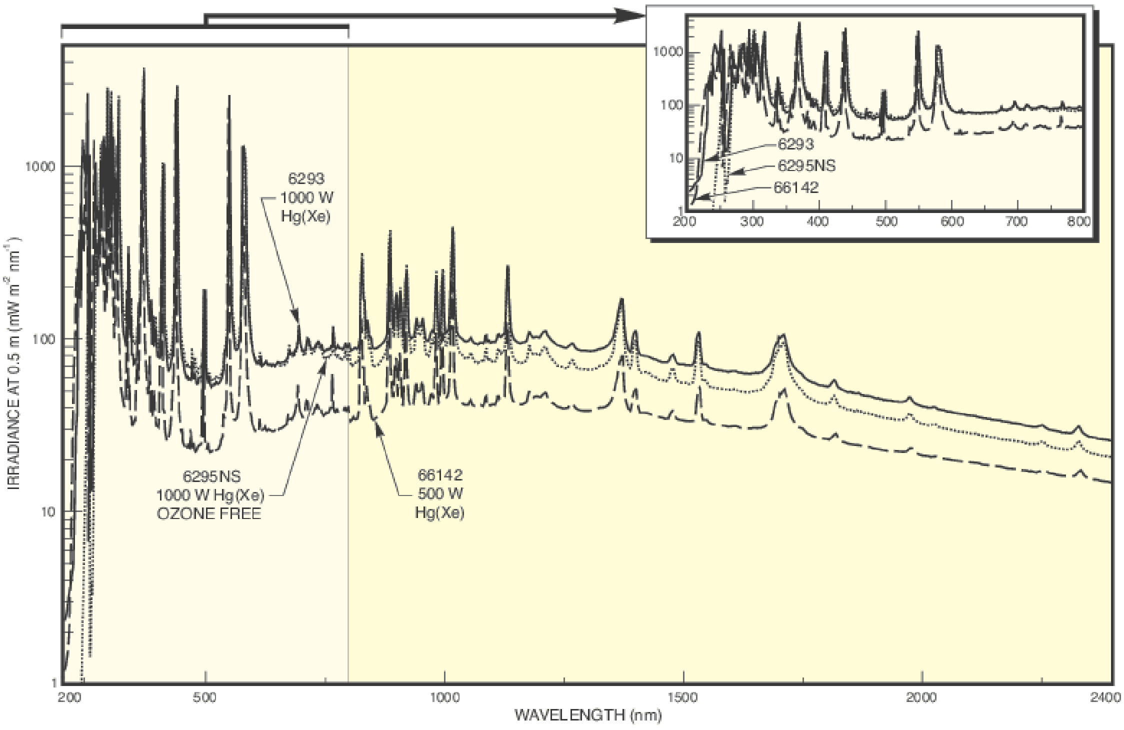

Figure S1. Spectrum of high power Mercury-Xenon Hg (Xe) lamps used in this study, photo reproduced from [1].

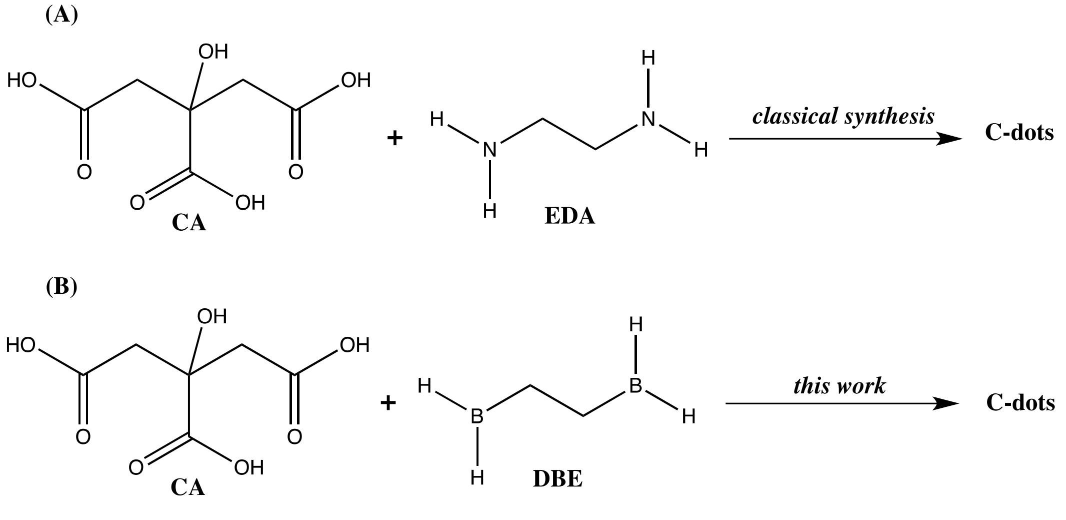

Figure S2. Schematic illustration of C-dots synthesis: (**A**) classical nitrogen-doped C-dots synthesis from CA and EDA; (**B**) "boron-doped" C-dots synthesis from CA and DBE in this work.

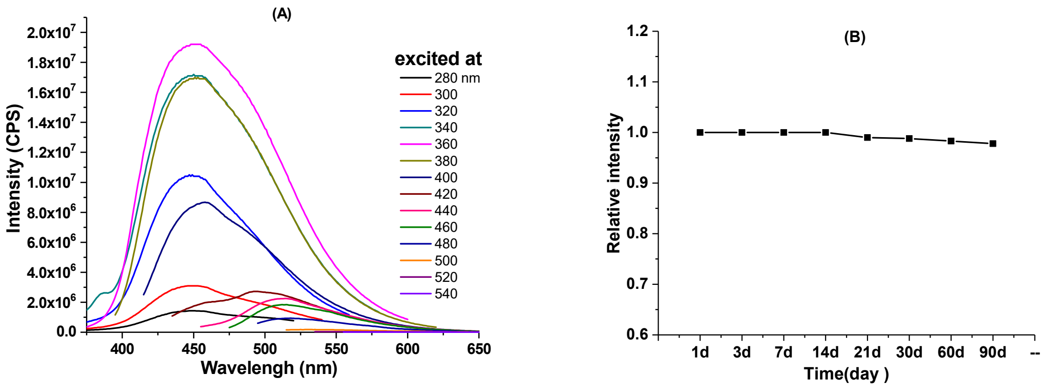

Figure S3. (**A**) Fluorescence emission spectra of C-dots excited at different wavelengths; (**B**) relative fluorescence intensity of C-dots exposed to ambient light with different time durations.

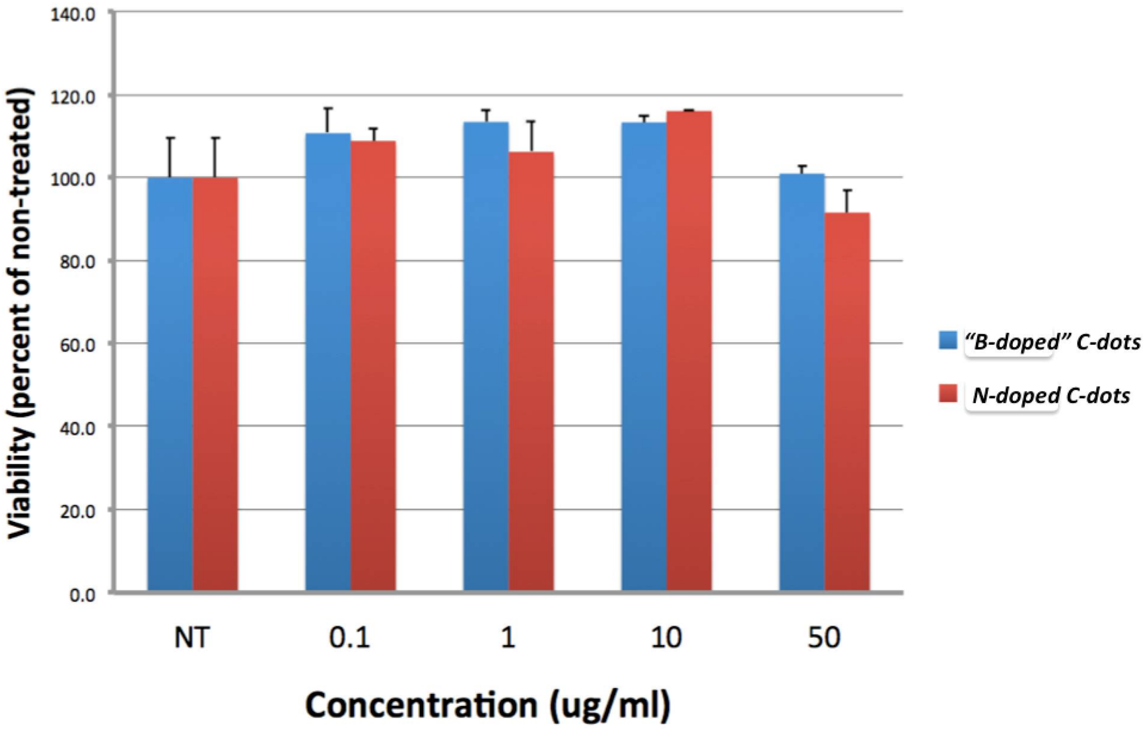

Figure S4. Cell viability test of C-dots using MTS assay: cell viability of mesenchymal stem cells treated with various concentrations of "B-doped" C-dots from CA and DBE prepared in this study (blue column), and N-doped C-dots synthesized from CA end EDA (red column), respectively.

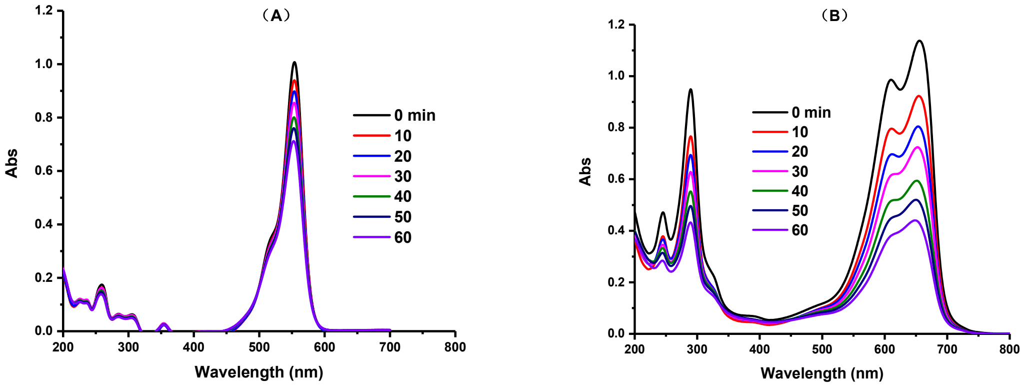

Figure S5. The photo-degradation of RhB **(A)** and MB **(B)** without the presence of photocatalysts. Our group has studied the degradation of RhB and MB alone under exact same condition as in this study, and the corresponding data has been reported by our group [2]. To demonstrate the efficiency of "B-doped" C-dots, the data is provided here for comparison purpose.

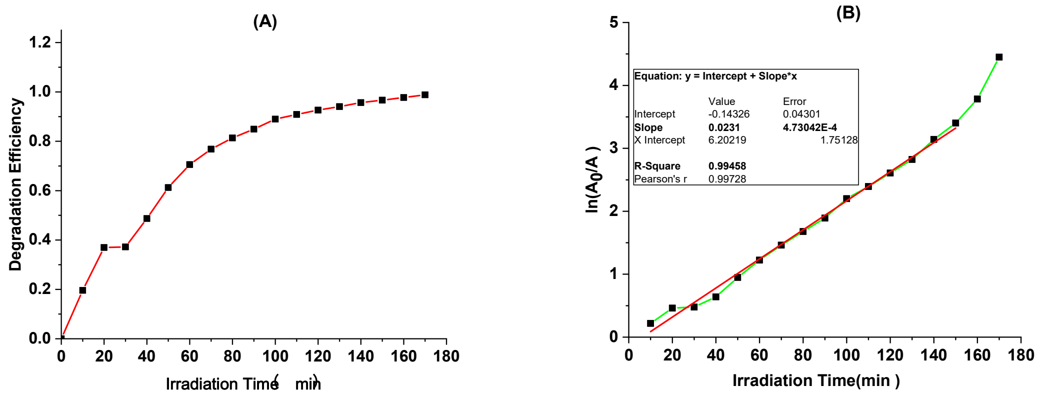

Figure S6. The photocatalytic degradation of MB catalyzed by C-dots (0.75 mg/mL): (**A**) the degradation efficiency (%) of MB by monitoring the absorption changes at 664 nm at different irradiation time; (**B**) irradiation time-dependent pseudo first-order kinetics plot (ln(A_0/A) vs. irradiation time) of MB.

Table S1. Table showing the XPS elemental atom % and BEs of O 1s, N 1s and C 1s core levels.

"B-doped" C-dots		
Orbitals	Atom (%)	BE peaks (fwhm, % integrated peak area)
O 1s	19.1	530.9 eV (2.4, 100%)
N 1s	5.1	399.3 eV (2.8, 100%)
C 1s	75.8	284.9 eV (2.6, 74.7%), 286.8 eV (2.1, 25.3%)

X-ray photoelectron spectra (XPS) were acquired using a Perkin-Elmer PHI 560 (Waltham, MA, USA) system with a double-pass cylindrical mirror analyzer operated using a Mg Kα anode with a hν = 1253.6 eV photon energy operated at 250 Watts and 13 kV. "B-doped" C-dots samples were mounted as a paste onto a custom Ta foil sample holder, and inserted into the XPS system via turbopumped antechamber. No signal was observed from the Ta 4f orbitals, indicating that the foil sample holder was completely covered. The observed C 1s core level at 284.9 eV emanating from the C=C alkenyl "B-doped" C-dot samples as previously measured in our laboratory [3] was used as a binding energy (BE) reference. Core level intensities of the O 1s and C 1s orbitals were normalized

using their known atomic sensitivity factors [4]. XPS peaks were curvefitted using 70% to 30% Gaussian-Lorentzian lineshapes with Shirley background subtractions [5]. BE peak envelopes were deconvoluted using CasaXPS ver. 2.2.107 (Devonshire, UK) software.

References

1. https://www.newport.com.cn/f/research-arc-lamp-sources-450-1000-w, accessed on July 28th, 2020.
2. Zhou, Y.; Zahran, E.M.; Quiroga, B.A.; Perez, J.; Mintz, K.J.; Peng, Z.; Liyanage, P.Y.; Pandey, R.R.; Chusuei, C.C.; Leblanc, R.M. Size-dependent photocatalytic activity of carbon dots with surface-state determined photoluminescence. *Appl. Catal. B: Environ*. **2019**, *248*, 157–166.
3. Zhou, Y.; Desserre, A.; Sharma, S.K.; Li, S.; Marksberry, M.H.; Chusuei, C.; Blackwelder, P.L.; Leblanc, R.M. Gel-like carbon dots: characterization and their potential applications. *Chem. Phys. Chem.* **2017**, *18*, 890–897.
4. Wagner, C.D.; Davis, L.E.; Zeller, M.V.; Taylor, J.A.; Raymond, R.H.; Gale, L.H. Empirical atomic sensitivity factors for quantitative analysis by electron spectroscopy for chemical analysis. S*urf. Interface Anal*. **1981**, *3*, 211–225.
5. Shirley, D.A.; High-resolution X-ray photoemission spectrum of the valence bands of gold. *Phys. Rev. B*. **1972**, *5*, 4709.
